# ‘Seeing the Dark’: Grounding Phenomenal Transparency and Opacity in Precision Estimation for Active Inference

**DOI:** 10.3389/fpsyg.2018.00643

**Published:** 2018-05-04

**Authors:** Jakub Limanowski, Karl Friston

**Affiliations:** The Wellcome Centre for Human Neuroimaging, Institute of Neurology, University College London, London, United Kingdom

**Keywords:** active inference, attention, mental action, opacity, transparency, self-model

## Abstract

One of the central claims of the Self-model Theory of Subjectivity is that the experience of being someone – even in a minimal form – arises through a transparent phenomenal self-model, which itself can in principle be reduced to brain processes. Here, we consider whether it is possible to distinguish between phenomenally transparent and opaque states in terms of active inference. We propose a relationship of phenomenal opacity to expected uncertainty or precision; i.e., the capacity for introspective attention and implicit mental action. Thus we associate introspective attention with the deployment of ‘precision’ that may render the perceptual evidence (for action) opaque, while treating transparency as a necessary aspect of beliefs about action, i.e., ‘what I am’ doing. We conclude by proposing how we may have to nuance our conception of minimal phenomenal selfhood and agency in light of this active inference conception of transparency-opacity.

## Phenomenal Transparency-Opacity of World- and Self-Models

The Self-model Theory of Subjectivity (SMT; Metzinger, 2003, 2004, 2007, 2008, 2013, 2014, 2017) is an exhaustive proposal for how and why consciousness is subjective, and how the epistemic subject and the phenomenal self are related. In this paper, we discuss how SMT’s central claims may be accommodated by the active inference framework, i.e., how we may conceive of a possible distinction of phenomenally transparent or opaque mental states, and which more general implications this has for our conception of self-models in the active inference formulation. Our effort is motivated by SMT’s explicit endorsement of a possible reductive identification of phenomenal self-models (PSMs) in the human brain and its affinity to ‘predictive processing’ accounts of brain function (Limanowski and Blankenburg, 2013; Wiese and Metzinger, 2017). We begin by presenting a brief overview of SMT’s key concepts, which we later link to active inference.

First, it is important to emphasize that SMT itself is almost exclusively concerned with *conscious* mental states, like for instance the experience of being someone – even if (or especially if) those conscious experiences are pre-reflective and non-conceptual. As a representationalist account, SMT subscribes to the assumption that what is consciously experienced is the representational content of a certain mental state realized by a certain carrier. SMT thus sets out to explain why we feel like we are in immediate touch with the world, although our very experience arises through operation on mental representations. SMT proposes that this is because the corresponding mental representations are phenomenally *transparent* to us. Transparency is a concept with some tradition that can metaphorically be understood as looking through a window onto the world, instead of looking *at* the window itself: we only access the representation’s intentional content (something in the world which it is about) without noticing its non-intentional carrier properties (cf. e.g., Moore, 1903; Harman, 1990; Tye, 1999; Lycan, 2015). Metzinger (2008) extends the traditional transparency notion by the claim that not (only) the carrier, but the ‘construction process’ of a phenomenally transparent mental representation is inaccessible to *introspective attention* – an inward-directed, sub-symbolic resource allocation within representational space (Metzinger, 2003). Conversely, if its construction process *is* accessible, this particular mental representation will be phenomenally *opaque*. Thus, whereas the content of a transparent representation is experienced as something mind-independent in the world, phenomenal opacity is the converse experience of some mental content *as being constructed* by one’s mind. Examples of opacity are thoughts (deliberately creating and using abstract representations), ‘lucid’ dreams (being aware that one is currently dreaming), or pseudo-hallucinations – whereby there can be gradual changes in transparency-opacity (Metzinger, 2003). An important property of such a phenomenal transparency-opacity gradient is that it is a marker of ‘epistemic reliability’; i.e., it reflects my subjective certainty that my experience is or is not made up by my mind (Metzinger, 2013, 2014). Note, however, that whether an experience has this marker of epistemic reliability (i.e., it feels real) is independent of whether or not the experienced content is veridical (e.g., hallucinations can feel very real without being veridical, Metzinger, 2014) and likewise independent of the cognitive availability of the knowledge that mental contents are based on representational processes. I.e., we may know or believe that some mental content is based on an inner construction process – we may, for example, acquire such a belief or knowledge while reading Metzinger – but this does not mean that the construction process itself is introspectively accessible to us (cf. Weisberg, 2005). In other words, “one cannot ‘think oneself out of’ one’s phenomenal model of reality with the help of purely cognitive operations alone” (Metzinger, 2003, p. 357), which implies that phenomenal transparency-opacity is ‘cognitively impenetrable’ (cf. Fodor, 1983)^1^. Together, these properties make opacity one of our mind’s most valuable tools, as it allows us to operate on representations that *we know* can be misrepresentations (Metzinger, 2003).

Crucially, SMT builds on phenomenal transparency as an essential condition for phenomenal selfhood: just as a transparent world-model grants the experience of being in immediate touch with a world, a transparent *phenomenal self-model (PSM)* affords the experience of being in immediate relation to a self. By ‘hiding’ the representational process underlying the PSM, transparency lets the system – you – experience just the content of its PSM, e.g., a self-location inside a body with an associated geometrical sensory perspective. Thus, transparency leads to identification of the system with the content of its PSM (Metzinger, 2003, 2004). This instantiation of phenomenal selfhood enables all higher forms of self-awareness. Most importantly, the content of a transparent PSM can be used as the ‘subject component’ in a phenomenal model representing the directedness of the subject (my consciously experienced ‘self’) to a consciously experienced ‘object component’. This co-representation of the self and the intentionality relation produces the experience of a ‘strong’ first-person perspective that makes consciousness subjective (Blanke and Metzinger, 2009). If, finally, such a model is (transparently) supplied with the capacity to select specific portions of representational space by directing its introspective attention, the system experiences ‘attentional agency’ and experiences itself as an epistemic, ‘knowing’ self – the PSM has become an *epistemic agent model* (EAM, Metzinger, 2008, 2013; cf. Graziano and Webb, 2015).

After this necessarily brief introduction, and in the hope that “a reductive identification of the PSM is possible” (Metzinger, 2008), we will offer a description of processes realizing^2^ phenomenally transparent or opaque mental states in the active inference formulation. This will have implications for how we may or may not conceive of a possible implementation of PSMs, to which the transparency-opacity distinction is fundamental.

## Phenomenal Transparency-Opacity as Precision Estimation for Active Inference

Active inference can be situated within a larger ‘free-energy principle,’ along which any living system will actively try to remain in a set of unsurprising states by performing inference; i.e., model selection and inversion (Friston, 2010; cf. Hohwy, 2013; Clark, 2015). The models in play here are *generative models* – probabilistic (predictive) mappings from causes (e.g., latent or hidden states of the world) to consequences (e.g., sensory observations, Friston et al., 2017a). If stacked, they yield a deep or hierarchical generative model (HGM), in which higher levels contextualize lower levels, and lower levels provide evidence for higher levels. In this scheme, free energy minimization corresponds to maximizing Bayesian model evidence, which implies a notion of ‘self-evidencing’ (i.e., a Bayes-optimal model – a free energy minimizing agent – will always try to maximize evidence for itself, Hohwy, 2016; Kirchhoff et al., 2018). The HGMs of interest in this paper are implemented in the human brain (for the detailed neuroanatomical implementation, see Bastos et al., 2012; Friston et al., 2017a,b,c; Parr and Friston, 2017a,b).

Under a popular algorithmic scheme known as predictive coding (Srinivasan et al., 1982), free energy (‘surprise’) is approximated in the form of precision-weighted prediction error signals, which are passed from lower to higher levels to update the model’s ‘beliefs’ about its environment, which in turn issue predictions to suppress prediction errors in lower levels (Friston, 2010). Note that we use the term ‘belief’ to refer to a conditional expectation (i.e., probabilistic representation) encoded by neuronal activity, rather than in the folk-psychological sense. This hierarchical scheme of recurrent message passing notably implies that increasingly higher-level beliefs represent increasingly abstract states of the environment at increasingly broad time scales. In such deep architectures, balancing the relative dominance of prior beliefs or sensory evidence (i.e., prediction errors) across the entire hierarchy is accomplished by weighting the ascending prediction errors by their precision. This means precision has to be estimated and deployed (at each level of the hierarchy): a process that is equated with attention (Feldman and Friston, 2010). Perceptual inference, under these principles, associates conscious experience with the ‘explanation’ for the sensorium that minimizes prediction error throughout the hierarchy (Hohwy, 2013; Seth et al., 2016).

### Planning and Selecting Optimal Actions

*Active* inference extends this scheme by noting action offers another way to minimize prediction errors or surprise; i.e., sampling sensory data in a way that corresponds to the model’s beliefs. Acting thus involves both generating a prediction of the sensory input expected to result from intended movement, and ‘fulfilling’ this prediction by executing the movement, thus effectively suppressing a prediction error signal that would otherwise emerge (Friston et al., 2010; Adams et al., 2013a; Brown et al., 2013; Seth and Friston, 2016).

Crucially, active inference must also entail the *selection* of optimal actions, i.e., it must include beliefs about which course of action will be optimal in a given context. Hence the agent’s model must be able to entertain ‘counterfactually rich’ representations; i.e., beliefs about several alternative *potential* actions and the states of affairs that these actions would bring about (Friston et al., 2014, 2017c; Seth, 2014; Pezzulo, 2017; cf. Powers, 1973/2005). This issue has recently been addressed by a formulation of an active inference in terms of minimizing expected free energy under different courses of action (Friston et al., 2017b,c; Parr and Friston, 2017a,b). This is formally identical to Bayesian model selection among potential courses of action (i.e., policies), based on their expected free energy (evaluated in the light of prior beliefs and preferences, Parr and Friston, 2017b). Policy selection thus entails selecting a sequence of actions, whose effects on state transitions will be more or less precise.

This has an important implication, from which much of our argument follows: policies necessarily entail a specification of the precision of (action-dependent) changes in hidden states that we are trying to infer. Formally, this means policies or beliefs about action *entail* expectations about precision. Therefore, expectations about precision are an inherent part of a policy. Heuristically, this means placing confidence in the consequences of action is an inherent part of the policies from which we select our actions. This implies that we cannot *place* confidence in our policies because this would induce another policy (of policies) and an infinite regress^3^. In what follows, we will consider the deployment of expected precision or confidence, entailed by policy, in terms of introspective attention and ‘mental’ action.

### Opacity, Attention, and Precision

Recall that according to SMT, the defining characteristic that disambiguates phenomenally transparent and opaque representations is that the construction of opaque representations is amenable to *introspective attention*, an inward-directed form of resource allocation onto specific parts within my internal reality-and-self-model (Metzinger, 2003). This distinction affords a simple formulation in terms of active inference, where attention is mediated by assigning greater or lesser precision to prediction errors at various levels of hierarchical processing. Importantly, this precision itself has to be predicted; implying that we have (first-order) representations of the (second-order) precision of hierarchically subordinate prediction errors. From the perspective of predictive coding, this means that we also have to infer the deployment of precision which, in a hierarchical setting, starts to look like attention (Feldman and Friston, 2010). In enactive formulations of predictive coding (i.e., active inference), descending predictions prescribe action. In terms of descending (first-order) predictions of content, this is usually cast as controlling motor (and autonomic reflexes) through descending proprioceptive or interoceptive predictions, respectively (Adams et al., 2013a; Seth and Friston, 2016). However, we can apply exactly the same principles to *descending predictions of precision* and thereby understand the active deployment of precision weighting as a form of ‘mental action’ that has exactly the look and feel of introspective attention. This argument rests on an assumed similarity of introspective and ‘perceptual’ attention (as implied by most transparency accounts, e.g., Harman, 1990; Metzinger, 2003); consequently, introspective attention is seen as a special case of the general mechanism of precision estimation applied to conscious mental representations. In this sense, there is a nice wordplay one could engage in following the etymology of ‘opacity’ (from Latin *opacitas*, from *opacus* ‘darkened’); i.e., that we can ‘see the dark’ – where holding beliefs (i.e., posterior expectations) about our very low precision enables phenomenal opacity.

The notion that our capacity to entertain opaque percepts depends upon expectations of precision has some interesting – and possibly fundamental – implications for perceptual inference. In particular, it means that part of our inference comprises (first-order) expectations about (second-order) precision or confidence. The question then arises (cf. Metzinger, 2017) what sorts of representations are the subject of mental actions, i.e., which prediction errors are subject to introspective attention. The answer clearly depends upon the architecture of the generative model. In terms of perceptual inference about states of the world, attention would be manifest either endogenously or exogenously in terms of precision weighting at intermediate and lower (sensory) levels of hierarchal processing, respectively. This implies that the associated perceptual beliefs or representations, by nature of their construction, have the capacity to lose phenomenal transparency and become opaque – simply because they are the product of introspective attention. One might now ask whether all precision weighting constitutes a form of mental action. If mental action is the deployment of top-down precision control then the answer would be no. This is because local optimization of precision (e.g., those processes associated with neurobiological adaptation and contrast gain control) does not necessarily entail any descending predictions of precision from higher levels. Put simply, mental action – in this setting – is just the broadcasting of predictions of precision to lower levels, where these predictions constrain and select ascending prediction errors.

The argument presented here speaks to SMT, in that it links opacity to the deployment of introspective attention (precision) to any hierarchical processing level currently informing a certain belief (a percept or a concept). Note that we are concerned with the predictive top-down deployment of precision – not with the precision of the high-level belief (perceptual content) itself; this fits nicely with the fact that increasing attention to a percept – a hallucination, for instance – does not necessarily make it appear more or less ‘mind-independent.’^4^ Further, on this view opaque representations require computational effort; i.e., they bind attentional resources, which opens up an interesting link to experiences like being ‘lost in thought’ or vivid mental imagery (e.g., Schooler et al., 2011). It also suggests that opacity, due to its computational cost, is not the default operating mode of (conscious) inference, and that this remarkable capacity has developed quite late (phylo- and ontogenetically, Metzinger, 2003, 2008). In other words, we can assume that conscious states resulting from inference have the phenomenal quality of transparency per default.

There are cases in which sensory evidence can trigger a loss of transparency – i.e., a revision of beliefs about precision. Such subjectively surprising changes from transparency to opacity encompass, for instance, reaching ‘lucidity’ in a dream (i.e., becoming aware that one is dreaming), or certain stress situations; e.g., after accidents, when somehow everything about the situation seems ‘unreal’ (Metzinger, 2003, 2008). I can, of course, also act to test whether some percept is ‘real’; such as when I recognize a visual percept as an afterimage because its position is not invariant to my eye movements. The latter case fits well with Seth’s (2014) related proposal that the perceived ‘realness’ of objects depends on the identification of features invariant to some potential (counterfactual) manipulation of the object.^5^ Accordingly, if I cannot form a counterfactually ‘rich’ model of an object, i.e., if I cannot encode a range of sensorimotor expectations about it, this object will seem ‘unreal’ to me (Seth, ibd.). Here, an interesting link to our proposal is that counterfactual richness of sensorimotor expectations is determined by the ‘temporal thickness’ of the planning process, as discussed in the next section.

In more dramatic cases, inference about precision may itself be compromised and produce abnormal experiences; e.g., in derealization or depersonalization (cf. Metzinger, 2003; Seth et al., 2012) or altered states of consciousness (Millière, 2017). These conditions may provide further insight into predictive coding formulations of the transparency-opacity distinction as resting upon introspective attention and posterior beliefs about the precision of perceptual prediction errors. On this view, derealization and related phenomena (such as hallucinosis, Friston, 2005) can be described in terms of a pathological or pharmacologically induced change in the precision of perceptual prediction errors (e.g., through the use of psychedelic drugs). Because this precision is itself inferred, there will be a necessary belief updating about precision at higher levels that may destroy phenomenal transparency; leading to an experience that departs radically from prior beliefs. Technically, predictive coding will drive posterior expectations about precision at higher levels in a way that violates prior expectations about the precision or attentional salience that would usually be afforded a sensory stream. This theme of aberrant precision control dominates many explanations of false perceptual inference in general – not only related to self-experience – and lack of central coherence in psychiatric syndromes such as organic psychosyndromes (Collerton et al., 2005), functional motor symptoms (Edwards et al., 2012), autism (Pellicano and Burr, 2012; Van de Cruys et al., 2014), and schizophrenia (Adams et al., 2013b; Powers et al., 2016, 2017; interestingly, derealization and depersonalization are often concomitants of the prodromal phases of psychosis). However, we submit that the above cases should be seen as rare exceptions, in which inference underlying the generally evolutionary beneficial ability to operate on opaque representations has gone wrong.

In sum, the key implication of the above is that the phenomenological analysis of opacity can be supplemented by the formal (mathematical) analysis of active inference. If one subscribes to the definition of opacity as the capacity to infer or predict the precision or confidence afforded a percept, several key questions about phenomenal transparency-opacity and its relationship to phenomenal selfhood and agency can, in principle, be addressed. In what follows, we will offer some answers to these sorts of questions in terms of the hierarchical deployment of predictive precision.

## Implications for Models of Minimal Selfhood and Agency

As indicated above, there is one fundamentally important set of posterior beliefs that are both privileged and impoverished – in the sense that they can never be subject to introspective attention. These are the beliefs about policies or sequences of (overt and mental) action that gather evidence from lower (perceptual) levels of hierarchical processing, because expectations about precision are an inherent part of a policy. Heuristically, this means placing confidence in the consequences of action is an inherent part of the policies from which we select our actions. This implies that we cannot, literally, *place* confidence in our policies. In other words, if the genesis of expectations about precision is, in and of itself, entailed in a (mental) action, *beliefs about action cannot be subject to introspective attention*. In other words, posterior beliefs about action are causes, not consequences, of introspective attention (and other actions). This suggests that beliefs about ‘what I am’ doing are unique, in the sense that they are necessarily transparent. This fits comfortably with SMT – in that these beliefs are inherently about the self and how the self is acting on the world.

One can unpack this argument further and identify examples of transparency that, in virtue of being prescribed by overt or covert action – can never become opaque. A nice example of this is the deployment of sensory precision during sensory attenuation (Brown et al., 2013; Limanowski, 2017; Wiese, 2017a). This is the converse of attention and an important aspect (on the current account) of mental action. For example, I cannot reverse saccadic suppression and manipulate the perception of optic flow during saccadic eye movements. I.e., active inference prescribes that if I commit to making a specific saccadic eye movement, I am also committed to saccadic suppression and the attenuation of prediction errors reporting optical flow during this eye movement. This commitment is entailed by the selection of the hypothesis ‘I am making a saccade’ and cannot be reversed until I select another action. Having executed the saccade, I am then committed to attending to the sensory impressions I have chosen to sample (e.g., to the ‘redness’ of the tomato) in a way that precludes attenuation of the relevant prediction errors. In short, beliefs about action whether overt or covert (attentional) are necessarily transparent – and are realized by active sampling of the sensorium that has transparency ‘written into it.’

In other words, our argument is that a minimal sense of agency and selfhood *cannot* be rendered opaque in the way described above, because beliefs about my action are necessarily transparent. I.e., although we can recognize that the sensory evidence for what we are doing may violate prior expectations, beliefs about what we are doing and how we are actively engaging with the world remain unshaken – because it is these beliefs that generate the prior points of reference that enable us to experience our perception in an opaque fashion in the first place. In the following, we sketch how this argument may shed new light on previous conceptions of ‘selfhood’ within active inference.

In the last couple of years, several proposals of ‘minimal’ self-model implementations under a predictive coding scheme have been advanced (Seth, 2013; Limanowski and Blankenburg, 2013; Limanowski, 2014; Apps and Tsakiris, 2014; Ishida et al., 2015; Allen and Friston, 2016). These proposals have in common that they treat the ‘self’ as a hypothesis within the HGM, which tries to maximize its evidence by minimizing prediction errors (via updating beliefs or by acting on the world). By the hierarchical nature of the underlying predictive coding architecture, these accounts have impressively explained the ‘centeredness’ of the model onto the ‘self,’ and why higher levels of the (self-) model will be increasingly abstract, complex, and invariant; i.e., these high-level self-representations will be less likely to be affected by prediction error. The idea of a hierarchy of ‘self’ priors resonates with the often proposed idea of a non-conscious and bodily basis for higher forms of self-consciousness (Gallagher, 2000; Gallese and Metzinger, 2003; Butz, 2008).

The notion of ‘control as inference’ as presented here (i.e., minimizing expected free energy under different courses of action) affords a new view on these proposals. Notably, the representation of several possible policies for scenarios in such a scheme – each specifying an expectation of how the state of the world unfolds depending on my action – implies an explicit representation of (fictive) time (Friston et al., 2017a). Depending on how far into the future (and the past) this representation of fictive time extends, potential action policies will be temporally deeper or ‘thicker.’ Recently we have proposed an association of this temporal depth or ‘thickness’ of policies – the ability to plan and explore several possible futures using a ‘thick’ model of time – with the degree of consciousness it subtends: whereas non-conscious processes are stuck in the ‘here-and-now’ (Edelman, 2001), conscious processes operate under a ‘thick’ model of the future with distant temporal horizons (Friston, 2017). This speaks to definitions of consciousness as a memory-dependent process of ‘protension’ or ‘mental time travel’ in embodied agents (James, 1890; Damasio et al., 1996; Edelman, 2003; Seth, 2009; Damasio, 2012; Verschure, 2016; Wiese, 2017b). Note that this links the degree of consciousness to the temporal thickness of active planning (as inference), not the depth of perceptual inference. The temporal *stability* implied by hierarchical depth (cf. Seth, 2009; Dehaene et al., 2017) should therefore be distinguished from temporal *thickness* in the sense implied by active inference as planning. Whereas temporal stability increases hierarchically via increasingly broad time scales, temporal thickness describes how far into the future (and the past) I can project *my course of action* and its consequences, which can – in principle – operate on various (fictive) time scales. Although longer time scales at high levels of inference may imply a projection into a farther future (cf. Hobson et al., 2014), the crucial point is that temporal thickness relates to an inference problem about control (my action), not about temporal invariance of the world.

Following this conception of active inference as planning, the modeling or hierarchical filtering processes described in the above predictive coding accounts of the self can now plausibly be conceived of as providing evidence for competing action policies – and thus inform Bayesian model selection processes that realize temporally thick planning. In turn, the selected policy will specify empirical priors that contextualize predictive coding at lower levels. To illustrate, it may help to refer to recent formulations of visual exploration using mixed (continuous and discrete state space) HGMs, in which the inferred eye position defines an additional hidden state that restricts possible future states and hence policy search (Friston et al., 2017b). Certainly, a ‘self-as-agent’ prior will be much more elaborate and its restrictions more profound, and – if we assume that planning more complex actions relies on temporally thick models or deep policy searches – these processes should also be available to consciousness. Speculatively, one could relate this bidirectional exchange to the specification of a phenomenal ‘unit of identification’, i.e., the phenomenal property with which I currently identify (Metzinger, 2013): top-down priors would constrain hierarchical predictive coding self-representations, which would in turn provide model evidence to policy (Bayesian model) selection defining beliefs about self-action – this would resonate with the active inference notion of ‘self-evidencing’ (Hohwy, 2016).

Now, what can we say about opacity of self-models? Although there is good reason to assume that – like all conscious states – conscious self-representations will be transparent per default, self-representation is perhaps the most impressive example of our capacity to operate on opaque representations. This capacity enables us to reflect on ourselves and also on other selves, which is one of our essential distinctions from most (in its complexity, probably from all) other animals (cf. Metzinger, 2008). We should carefully distinguish these cases from the transparency of minimal selfhood as described above; note, for instance, that it is indeed much easier for us to render certain kinds of self-models opaque (e.g., I can revise my conception of ‘my social self’) than our (transparently experienced) minimal and bodily foundations – in fact, rendering minimal self-models opaque could have dramatic consequences ranging from pathological experience to complete destruction of the self-experience (Metzinger, 2004; Limanowski, 2017). However, in principle a distinction between transparent and opaque self-models also makes sense for active inference – there should be similar opacity (precision) expectations that enable a cognitive ‘distance’ from the inferred self-representation by presenting the fact that it is *itself* a process (construction) of inference. One could argue that this ‘distance’ is a necessary component of perspective taking; literally so, in terms of projective geometries (Rudrauf et al., 2017).

This view speaks to the use of our (thick) temporal models of action and agency in the service of inferring the intentions of others. Hitherto, we have focused on posterior beliefs about causes of action associated with phenomenal transparency and necessarily implying a *perspectival* construction of the world. This can plausibly be associated with minimal phenomenal selfhood (especially when this perspective includes predictions about our own body and interoceptive inference). A different quality of self-consciousness may be accompanied by the (active) inference mandated by attribution of agency; namely, ‘I did that’ or ‘you did that.’ If certain creatures can use the repertoire of policies that they deploy for action selection to explain the actions of others, then we arrive at the Bayesian formulation of the mirror neuron system (Kilner et al., 2007). This brings with it an extra inference problem; namely, the attribution of agency. In other words, if we use the same models to explain our own behavior and the observed consequences of another’s behavior, then we also have to carefully infer agency (in order to appropriately attenuate proprioceptive precision and preclude echopraxia, Friston and Frith, 2015). In sum, as soon as a generative model entertains the hypothesis of ‘other creatures like me,’ there must be a distinction between the consequences of action of self, versus action of another. One might tentatively suggest that more elaborate forms of self-consciousness rest upon the capacity to entertain hypotheses that (the consequences of) action can plausibly be ascribed to another – leading to the capacity for perspective taking and theory of mind.

## Conclusion and Outlook

We have presented a possibility to distinguish between phenomenally transparent or opaque states within the formulation of active inference; by suggesting transparency is a necessary aspect of beliefs about action that entail introspective attention, while the precision expectations that underwrite introspective attention have the capacity to render the perceptual evidence (for action) opaque. Our argument resonates with SMT in taking transparency as the default presentation mode of conscious experience, while opacity involves the deployment of additional computational resources; opacity as introspective attention within an internal construction process of representations that enables more elaborate forms of inference; and ultimately, the necessary transparency of minimal self-representations.

Our proposal should be taken as an initial attempt at accommodating insights from philosophical accounts of selfhood and subjectivity within active inference. Despite many open questions, conceiving of phenomenal transparency-opacity as resulting from inference in temporally thick generative models allows new ways of asking questions about empirically tractable phenomena like self-reflection, metacognition (Schooler et al., 2011), and self-deception (Dehaene et al., 2017; Pliushch, 2017; Limanowski, 2017). It may help to understand the neuronal basis of pathologically or drug-induced altered states of experience (Seth et al., 2012; Millière, 2017), or lucid dreaming (Windt, 2010; Metzinger, 2013; Hobson et al., 2014) in terms of wrong-gone precision-weighting. With the recent rise of virtual reality tools (Sanchez-Vives and Slater, 2005; Suzuki et al., 2017), there are new ways of experimentally inducing ‘updates’ of opacity beliefs that, combined with brain imaging, could be used to specifically test some of the speculations advanced here in terms of their neuroanatomical implementation. Such an understanding may also guide the development of synthetic consciousness (Verschure, 2016).

Certainly, more conceptual analysis is needed. In the formulation endorsed here, we have assumed a general neuronal mechanism, which for *conscious* processes of inference (sufficient temporal thickness in planning) generates the phenomenology of transparency-opacity, but also underpins non-conscious inference. Although SMT fully acknowledges the role of sub-personal and non-conscious processes in grounding (self) models^6^, it has been objected that it ‘makes too much of the system phenomenal’ (Weisberg, 2005). Along these lines, the active inference formulation suggests that phenomenal transparency has to be seen as a default property of conscious states – including self-consciousness – arising from a general neuronal mechanism of model optimisation under temporally ‘thick’ inference, where some beliefs simply *cannot* be rendered opaque due to the model’s architecture. Conveniently, the active inference formulation thus accommodates SMT’s proposal of a limit to the mental self-modeling process that avoids an otherwise infinite regress.

Pushing our argument further, we presume that any fully conscious living system will develop conscious, transparent mental representations of its ‘self.’ Along active inference, there is already some minimal notion of selfhood entailed in the notion of a ‘self-evidencing’ model, using a statistical separation of self from non-self by a Markov blanket when consciousness arises. Our argument is further that consciousness will always go hand-in-hand with self-consciousness.^7^ At first sight, this may seem to contradict SMT’s claim that there can be phenomenal world-models without phenomenal self-models (Metzinger, 2003, 2004) and the related idea of temporally un-extended ‘minimal’ self-consciousness (Gallagher, 2000; cf. Blanke and Metzinger, 2009) – these points remain to be clarified. Finally, active inference as planning, due to its inherently ‘epistemic’ notion and the implied importance of ‘self’ priors, seems to us most closely related to the concept of an EAM (see above; Metzinger, 2013), which in SMT is *one* particular kind of PSM, transparently equipped with attentional agency. Since we assume transparency as a default presentation mode of consciousness, and attention as a universal mechanism across the entire HGM, perhaps we will have to conceive of *any* conscious self-model as an EAM.

## Author Contributions

All authors listed have made a substantial, direct and intellectual contribution to the work, and approved it for publication.

## Conflict of Interest Statement

The authors declare that the research was conducted in the absence of any commercial or financial relationships that could be construed as a potential conflict of interest.

## Footnotes

1 Rather, one could speculate that phenomenal opacity may be a necessary condition for ‘cognitive penetrability’ – we thank a reviewer for this suggestion.

2 The exact mind-brain relation is beyond the scope of this paper. Here, we simply try to identify the relevant physical (brain) processes in a meaningful way (cf. Tye, 1999; Seth, 2016). This is not to dismiss the importance of subjective experience – on the contrary, we believe that insights from phenomenological analysis may help developing theories of brain function.

3 This should not be read as saying policies *per se* do not have a precision. It is perfectly possible to *have* precise or imprecise beliefs over policies. Indeed, the neuromodulator dopamine appears to have an important role in optimizing the precision of beliefs about policies (Friston et al., 2014). What we are saying here is that the precision of policies cannot be predicted, because this would require another policy to generate the predictions – and so on *ad infinitum*. This can be nicely linked to SMT’s idea that a transparent mental self-model avoids an infinite regress of self-modeling that would occur under opacity of the modeling process (Metzinger, 2004).

4 However, one could conceive of cases where (abnormally) increased attention may lead to a loss of transparency; one such case may be the inhibition of normal motor function by increased attention to movement execution (see Limanowski, 2017, for a detailed discussion).

5 For some fine conceptual differences between Seth’s sensorimotor notion and SMT’s phenomenal transparency conceptualization, see Metzinger (2014).

6 A proposal that similarly spans mental and non-mental levels can be found in the ‘somatic marker hypothesis’ (Damasio et al., 1996).

7 Here we refer to self-consciousness as the basic experience of being a self (Blanke and Metzinger, 2009), which can be the basis for, but not necessarily implies higher, cognitive levels of self-awareness or self-recognition.
